# Dynamics of specific antibodies in COVID-19 patients after recovery

**DOI:** 10.1017/S0950268822000528

**Published:** 2022-03-22

**Authors:** Zhi-Bo Deng, Feng Cheng, Yong Zhang

**Affiliations:** 1 Department of General Surgery, Jianli People's Hospital, Jingzhou, China; 2 Department of Clinical Laboratory, Jianli People's Hospital, Jingzhou, China; 3 Department of Nephrology, Jianli People's Hospital, Jingzhou, China

**Keywords:** Antibody, COVID-19, IgG antibody, positive rate, vaccines

## Abstract

The ongoing pandemic of coronavirus disease 2019 (COVID-19) caused by severe acute respiratory syndrome coronavirus 2 (SARS-CoV-2) has led to an unprecedented global public health crisis. The objectives of this study were to analyse the dynamic trend in specific antibodies in the serum of patients infected with SARS-CoV-2 within 12 months after recovery and to make a preliminary assessment of the protective effect of vaccination. Eighty-seven patients with confirmed COVID-19 who were admitted to our hospital from January to February 2020 were followed after recovery. Three-millilitre blood samples were collected for specific antibody detection at four time points: 1, 6 and 12 months after recovery and 1 month after vaccination. The changes in specific immunoglobulin G (IgG) antibody and total antibody levels over 12 months were analysed. Moreover, an independent comparison of the neutralising antibody levels of patients after vaccination with those of healthy medical staff after vaccination was performed to compare the inhibition rates of the neutralising antibody to the virus. No statistically significant difference in the sex distribution between groups was observed (*P* > 0.05). Older patients had a greater risk of developing severe and critical COVID-19 (*P* < 0.05). The percentages of subjects positive for IgG antibodies at 1, 6 and 12 months after recovery were 88.5%, 75.9% and 50.6%, respectively. The rate of IgG antibody conversion from positive to negative was not uniform across time points: the change was slow in the first 6 months but increased significantly in the last 6 months (*P* < 0.05). The positive rate of critically ill patients in the first 6 months was 100.0%. The trend over time in total antibody levels was similar to that of IgG antibody levels. Over 12 months, the sample/cut off value of total antibodies continued to decrease, while that of different disease severities was significantly different (*P* < 0.05). After vaccine administration, the total antibody level exceeded the detection level in the first month, which was independent of disease severity (*P* > 0.05). Significant differences were observed in the inhibition rate of the neutralising antibody against the virus in the disease group and the control group (*P* < 0.05). IgG antibody produced by patients naturally infected with SARS-CoV-2 has a duration of no less than 1 year, and the change trend graph of total antibody levels was the same as that of IgG antibody levels. Under vaccine stimulation, the positive rate of IgG antibody was as high as 100%, and the total antibody concentration reached the highest level, which was independent of disease severity. Neutralising antibodies following vaccination in patients who recovered from COVID-19 had a higher inhibition rate against SARS-CoV-2 than those of vaccinated healthy controls, indicating that these COVID-19 patients had a lower risk of reinfection and were better protected.

## Introduction

The ongoing pandemic of coronavirus disease 2019 (COVID-19) caused by severe acute respiratory syndrome coronavirus 2 (SARS-CoV-2) has led to an unprecedented global public health crisis. As of 26 October 2021, more than 243 million cases of SARS-CoV-2 infection and more than 4.9 million deaths have been reported across the globe [1]. The spread and rate of mutation of SARS-CoV-2 have been extremely rapid [2, 3].

There is evidence that first-line hospital staff and elderly people, especially those with underlying medical conditions, are at higher risk of death and have a worse prognosis in the case of infection with SARS-CoV-2 [4, 5]. The decrease in immune function associated with old age has been proven to be an important factor leading to the development of severe COVID-19 [6]. In the context of the novel coronavirus global pandemic, it is crucial to assess the potential role of new coronavirus vaccines in preventing COVID-19 disease [7].

Due to the lack of effective treatment drugs, the new coronavirus vaccines are one of the most effective ways to contain the spread of the disease. An inactivated coronavirus vaccine has been demonstrated to induce high neutralising antibody titres in mice, rats, guinea pigs, rabbits and animal primates [8, 9]. In addition, the results of previous clinical trials in many countries have shown that the recipients of the new coronavirus inactivated vaccine had a positive neutralising antibody response [10–13].

However, determining how long antibodies produced by COVID-19 vaccines last in the human body and how titre levels change over time require further study. To this end, 87 patients with confirmed COVID-19 admitted to our hospital from January to February 2020 were followed for 18 months. Various antibody levels in the patients were analysed to characterise the changes in specific antibodies and provide a theoretical reference for the prevention and control of COVID-19.

## Materials and methods

### General information

With the approval of the ethics committee of Jianli People's Hospital (20200103), 105 patients with COVID-19 hospitalised in our hospital from 20 January 2020 to 29 February 2020 were selected as the research participants. To improve the participation rate and reduce the number of dropouts, patients living in the urban area of our city were selected as much as possible for tracking, and the number of participants in the whole study was 87. There were 47 males (54.0%) and 40 females (46.0%), aged from 27 to 84 years, with an average age of 53.6 ± 12.6 years and an average treatment time of 21.4 ± 9.6 days.

### Diagnostic and recovery criteria

#### Diagnostic criteria

Suspected cases of SARS-CoV-2 infection were determined on the basis of the epidemiological history and clinical manifestations and one of the following aetiological or serological findings: (1) real-time fluorescence reverse transcription polymerase chain reaction detection of SARS-CoV-2 nucleic acid positivity; (2) viral gene sequencing highly homologous with the known SARS-CoV-2 sequence and (3) SARS-CoV-2-specific immunoglobulin M (IgM) antibody and IgG antibody positivity, specific IgG antibody changing from negative to positive or an IgG antibody titre in the recovery stage four times higher than that in the acute stage.

#### Clinical classification criteria

1. Mild type: the clinical symptoms were mild, and no pneumonia was observed on imaging.
2. Ordinary type: the clinical symptoms also included fever, respiratory symptoms and imaging manifestations of pneumonia.
3. Severe type: patient condition meeting any of the following criteria:
  - respiratory distress, defined as a respiratory rate ≥30 times/min;
  - resting oxygen saturation ≤93%;
  - PaO_2_/FiO_2_ ≤300 mmHg;
  - clinical symptoms gradually worsening, and pulmonary imaging showing that lesions had progressed more than 50% within 24–48 h.
4. Critical type: patient condition meeting one of the following criteria:
  - respiratory failure, defined as the need for mechanical ventilation;
  - shock;
  - other organ failure requiring intensive care unit monitoring and treatment.

#### Recovery criteria

- Normal body temperature for more than 3 days.
- Significant improvement in respiratory symptoms.
- Pulmonary imaging showing that acute exudative lesions had significantly improved.
- Two consecutive negative nucleic acid tests of sputum, nasopharyngeal swab or other respiratory tract samples (the sampling time was at least 24 h). Those who met the above conditions at the same time were deemed to have recovered and were allowed to be discharged from the hospital

### Inclusion and exclusion criteria

*Treatment group*: The inclusion criterion was patients diagnosed with COVID-19 during hospitalisation at our hospital from 20 January 2020 to 29 February 2020. The exclusion criterion was patients who had lived outside the country long term for whom post discharge test data could not be collected.

*Control group*: The inclusion criterion was healthy staff at our hospital. The exclusion criterion was people with chronic diseases such as hypertension, diabetes or hepatitis.

### Test items and methods

The SARS-COV-2 IgM/IgG antibody (IgG/IgM antibody) levels were assessed qualitatively by using the colloidal gold method (Nanjing Novizan Medical Technology Co. Ltd) following the National Instrument Injection protocol 20203400239.

The SARS-COV-2 total antibody levels (undifferentiated IgM and IgG antibody levels) were assessed quantitatively by using the magnetic particle chemiluminescence method following the National Instrument Injection Standard 20203400198. The Caris200 automatic chemiluminescence immunoassay analyser (Xiamen Wantakai Biotechnology Co. Ltd) was used, and a sample/cut off (s/co) value >1.0 was considered to be reactive.

The SARS-COV-2 neutralising antibody levels were measured quantitatively by using enzyme-linked immunoassay for protective assessment after vaccine administration (Shanghai Jino Biotechnology Co. Ltd; reagent batch no.: lot: X20210201, validity: EXP2022.2.3, Thermo Multiskan FC spectrophotometer colorimetry).

The vaccine administered to the study subjects was an inactivated COVID-19 vaccine produced by Wuhan Institute of Biological Products Co. Ltd, with delivery dates from March to May 2020.

All the tests were completed by senior technicians of our hospital laboratory, and the whole process was subject to strict quality control management.

### Experimental design

The participants were classified into three groups based on a clinical assessment of disease severity:

- mild group;
- ordinary group;
- severe and critical group.

Due to the small number of participants per group, the severe and critical patients were combined into one group.

IgG antibody and total antibody were detected at 1, 6 and 12 months after recovery and at 1 month after the administration of the second dose of the vaccine. Neutralising antibodies were measured in healthy medical staff of our hospital at 1 month after completing the vaccine administration schedule, and the inhibition rates of the virus were compared.

### Statistical methods

The data were sorted in Excel, and statistical software SPSS 25.0 and R software (version 3.6.3) were used for data analysis. Continuous variables conforming to a normal distribution are represented by *χ* ± *s*, and an independent-sample *t* test was used for intergroup comparisons. The count data were expressed as percentages and analysed by the *χ*^2^ test and Fisher's exact test. The repeatability of binary variables was analysed by a generalised estimating equation (GEE) model, and the repeatability of continuous numerical variables was analysed by a general linear model, with *P* < 0.05.

## Results

### Relationship between disease and sex and age

A total of 105 subjects were enrolled; 18 dropped out during the course of the study, so the dropout rate was 17.14%. A total of 87 subjects completed the whole monitoring period and were included in the results of the study. To analyse whether sex and age were correlated with the development of critical disease, the mild group and the ordinary group were combined into one group.

Table 1 shows that sex was not associated with the development of severe or critical disease (*P* = 0.344) and that age was significantly correlated with the development of severe and critical disease (*P* = 0.023), indicating that older individuals had a greater risk of developing severe and critical disease.

**Table 1.** Effect analysis of sex and age on disease

	Patients	Mild and ordinary groups	Severe and critical group	*χ*^2^	*P*
Sex					
Male	47	43	4	0.895	0.344
Female	40	34	6		
Age					
<40	19	18	1	7.514	0.023
40–60	41	39	2		
>60	27	20	7		

### Dynamic analysis of SARS-CoV-2 IgG antibody levels

The positive rate of IgG antibody as measured at three time points before vaccination is shown in Table 2. The positive rate of IgG antibody was 88.5% at 1 month after recovery, 75.9% at 6 months after recovery and 50.6% at 12 months after recovery. After vaccination, the positive rate of IgG antibody was 100%.

**Table 2.** IgG positivity rate in patients over time

Time	Disease group	Mild group	Ordinary group	Severe and critical group
1 month	87 (88.5%)	35 (87.5%)	32 (0.864)	10 (100.0%)
6 months	66 (75.9%)	30 (75.0%)	26 (70.3%)	10 (100.0%)
12 months	44 (50.6%)	18 (45.0%)	18 (48.6)	8 (80.0%)
After vaccination	87 (100.0)	40 (100.0%)	37 (100.0%)	10 (100.0%)

To study the influence and trend of sex, age, disease classification and recovery time on the negative conversion rate of IgG antibody, the GEE model was used. The results are shown in Table 3.

**Table 3.** IgG antibody conversion rate by subgroup and time point

Parameter	*B*-value	OR (95% CI)	*P*
Sex	−0.647	0.52 (0.22–1.24)	0.142
Over 60-year-old	−0.773	0.46 (0.12–1.75)	0.255
Aged 40–60-year-old	−0.647	0.52 (0.17–1.66)	0.271
Severe (critical) group	−1.897	0.15 (0.03–0.73)	0.019
Ordinary group	0.280	1.32 (0.55–3.16)	0.528
12 months	2.155	8.63 (4.44–16.79)	0.001
6 months	0.801	2.23 (1.34–3.7)	0.002

Sex did not significantly affect the negative conversion rate of IgG antibody (*P* = 0.142). Age also had no significant effect on the IgG antibody-negative rate (*P* > 0.05).

There was a significant difference in the negative conversion rate of IgG antibody between the severe and ordinary COVID-19 groups and the mild COVID-19 group (*B* = −1.897, *P* = 0.019). The negative conversion rate of IgG antibody in the ordinary COVID-19 group was 0.15 times higher than that in the mild COVID-19 group, indicating that IgG antibody lasted longer in the ordinary COVID-19 group.

At 6 months after recovery, the IgG antibody-negative rate was significantly different from that at 1 month after recovery (*B* = 0.081, *P* = 0.002). The conversion efficiency was 2.23 times that at 1 month after recovery. At 12 months after recovery, there was a significant difference between the patient group and the control group (*B* = 2.155, *P* = 0.001), and the conversion efficiency was 8.63 times that at 1 month after recovery. In conclusion, at 6 months after recovery, the rate of IgG antibody conversion increased substantially and was 3.87 times higher than that during the first 6 months (8.63/2.23 = 3.87). The prediction accuracy of the model was 75.01% (24 + 172)/261 = 75.01% as shown in Table 4.

**Table 4.** Accuracy of model prediction

	Categories of predictions		Total
	Negative	Positive	
The measured results			
Negative	30	21	51
Positive	13	116	129
Total	43	137	180

### Analysis of SARS-CoV-2 total antibodies

A general linear model was used to analyse the effects of covariates on the total antibody levels of the patients, and the analysis results are shown in Table S1 as follows:

1. The change in the total antibody levels differed significantly by time point (*F* (3, 81) = 3.781, *P* = 0.014).
2. Age was not significantly associated with the total antibody level of the patients (*F* (1, 83) = 0.023, *P* = 0.881), and the total antibody level of the patients did not differ significantly by disease severity (*F* (2, 83) = 2.241, *P* = 0.113).
3. Furthermore, there was no interaction between the time point and age (*F* (3, 81) = 0.193, *P* = 0.901). There was a significant interaction between the time point and disease severity (*F* (3, 82) = 3.349, *P* = 0.023). Thus, the trend in the total antibody level of the patients according to the time point differed with different disease severities.

Based on the above analysis results, the interaction effect between different time points and different disease severities was significant; therefore, the effect between time points and treatment methods needs to be further investigated.

As seen in Table S2, there were significant differences in the total antibody levels at four time points between the ordinary group and the mild group (*P* < 0.05), but there was no significant difference in the antibody levels between the ordinary and mild groups and the severe and critical group at 6 months (*P* = 0.882) or 12 months after recovery (*P* = 0.001). These findings indicated that the duration of antibody response in the severe and critical group was longer than that in the mild and ordinary groups and that the antibody level could quickly revert to the initial level (that at the first month after recovery) after vaccination, with no significant difference among the groups (*P* = 0.957).

**Fig. 1.**
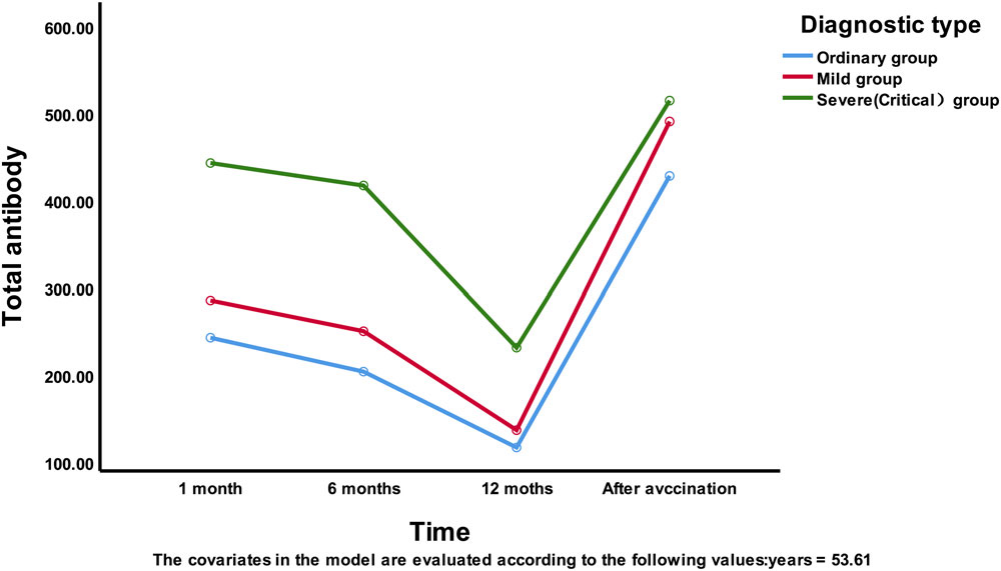
Trend chart of total antibody in patients.

As shown in Table S3, there was a significant difference in antibody levels between the ordinary group and the severe and critical group at only 6 months after recovery (*P* = 0.023). There was no significant difference in antibody levels among the different disease severity groups at other time points. Figure 1 illustrates the above results.

Figure 1 shows that the trend in total antibody levels over time was similar to the trend in IgG antibody levels. In the 12 months after recovery, the change was less pronounced in the first 6 months and more pronounced in the last 6 months. After vaccination, the antibody level increased rapidly. The total antibody concentration of the mild group was 3.64 times higher (489.12/134.51), the antibody concentration of the ordinary group was 3.70 times higher (427.04/115.35) and the antibody concentration of the severe and critical group was 2.18 times higher (518.06/237.84) after vaccination.

### Neutralising antibody analysis after vaccination

Both patients (experimental group) and medical staff at our hospital (control group) were administered two doses of an inactivated vaccine. Neutralising antibody concentrations were measured at intervals of 1 month after the completion of the vaccination schedule. The virus inhibition rates of the experimental group and control group were verified to have a normal distribution (*P* > 0.05). An independent-sample *t* test was used for analysis, and the results are shown in Table S4.

**Fig. 2.**
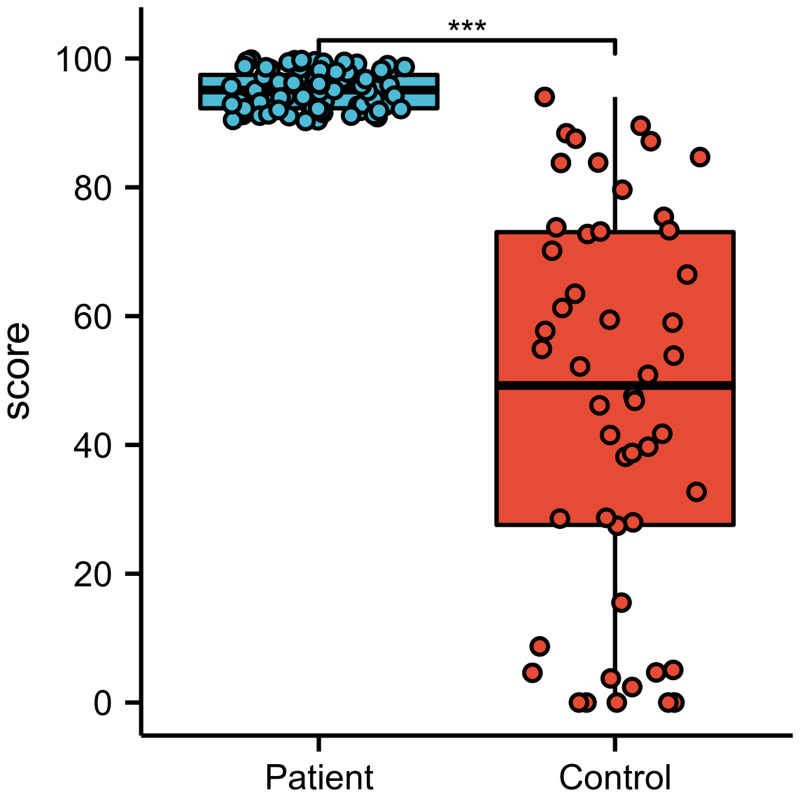
Comparison of the virus inhibition rate of neutralising antibodies.

According to the data in Table S4, there was no significant difference between the groups with respect to sex (*P* = 0.650) or age (*P* = 0.121). The neutralising antibody inhibition rates were significantly different between the groups (*P* = 0.001). As shown in Figure 2, the inhibition rate of the control group was significantly higher (2.04 times (95.01/46.53)) than that of the experimental group.

## Discussion

Inactivated vaccines are composed of virus particles, bacteria or other pathogens. The pathogens are grown under controlled conditions and are killed or inactivated in the process of culture. Inactivated viruses often elicit a weaker immune system response than live viruses, and multiple ‘enhanced’ administrations are needed to ensure an effective immune response to inactivated pathogens [14]. The antibody, which is specific, is produced by the immune system for defence after the body is infected with the virus. IgM antibodies are produced in the early stage of infection, and IgG antibodies appear after patients enter the recovery period. The presence of IgG antibodies can indicate the occurrence of a previous infection. There could be a potential delay in the production of antibodies following viral infection, which is of great importance for infection monitoring [15]. A study by Voysey *et al*. found that antibody titres peak 28 days after COVID-19 vaccination and that neutralising antibodies are produced by humoral immune responses that are effective at preventing reinfection when re-exposed to the novel coronavirus vaccine. In another study, subjects were administered inactivated vaccine injections twice, with an interval of 28 days, which repeatedly stimulated memory T cells and triggered their rapid proliferation and differentiation and the production of antibodies. The immune response to infection in the health personnel disease group, as measured by the virus inhibition rate, was approximately two times that of the control group, confirming that the risk of reinfection was very low [16].

A study by Bleier *et al*. showed that the risk of infection increased with a decrease in the antibody titre [17]. According to our data, the duration of IgG antibody levels in the experimental group was longer than that in the control group. One month after recovery, the IgG antibody positivity rate of the mild and ordinary groups was above 85.0% and that of the critical group was 100.0%. At 6 months, the IgG positivity rate of the severe and critical group was still 100.0%, which may be related to the long course of disease during the treatment period and the high viral load during the infection period [15]. After 1 year, the IgG positivity rate was approximately 50%, which indicates that the half-life of the antibody was approximately 1 year. The change trend was the same as that of SARS-specific IgG antibody, which reached its highest level in SARS patients 35 days after recovery and then began to decrease [18]. Wu *et al*. evaluated the antibodies of 176 recovered COVID-19 patients. The SARS-specific antibody lasted for 2 years on average and decreased significantly in the third year [19]. A 13-year follow-up found that SARS IgG antibodies could still be detected 12 years after infection, and the duration of SARS-CoV-2 IgG antibodies was significantly shorter than that of SARS IgG antibodies [20].

Our results found that the severity of disease in COVID-19 patients was not related to sex but was positively correlated with age, with older age groups showing a greater risk of severe and critical COVID-19, which is consistent with relevant reports in the literature [21, 22]. At 6 months after recovery, there was no significant difference between the observed IgG positivity rate and the results in the relevant literature [23], but the antibody levels observed in this study were slightly higher than those in the literature. This may be related to differences in the detection capabilities of various reagents (indirect method, capture method, double antigen sandwich method, etc.), immune markers (acridine, horseradish peroxidase, colloidal gold, etc.), the recombinant antigens used (receptor binding domain of spike protein, nucleocapsid protein, S1 protein) and different disease severities. Different detection reagents may be selective for different target antigens, which affects their detection efficiency [24–26]. In different stages of disease progression, the interaction between IgM and IgG antibodies and various target antigens differs, suggesting that when interpreting the results of assays that detect antibodies, we should keep in mind the role of different target antigens and disease stage [27].

The negative rate of IgG antibody at 6 months was 2.23 times higher than that at 1 month after recovery, and at 12 months, the negative rate of IgG antibody was 8.63 times higher than that at 1 month after recovery. At 6 months after recovery, the IgG antibody conversion rate was 3.87 times higher than that during the first 6 months (8.63/2.23 = 3.87), and the accuracy of the model prediction was as high as 75.01%. We assessed the s/co value of total antibody levels. The decrease in total antibody concentration in the first 6 months was minor, and the rate of decline increased in the last 6 months, which was consistent with the trend in IgG antibody concentration. The total antibody level is related to the course of disease. The total antibody concentration in the severe and critical group was higher than that in the mild and ordinary groups, and the rate of the decline in concentration was lower. After the completion of the vaccine administration schedule, the total antibody concentration increased rapidly to a level higher than that at 1 month after recovery and 2–4 times higher than the level before vaccination (12 months after recovery). As of April 2021, the experimental group had completed the COVID-19 vaccination schedule. Therefore, we cannot continue to observe the natural decay process of the antibody.

This study has the following limitations. (1) One subject with asymptomatic infection dropped out during study follow-up, and the results from that subject were not included in the analyses. (2) Only two subjects with critical disease were enrolled in this study. Although the patients with critical and severe cases were grouped together for analysis, the sample size of the combined group was still relatively small. (3) During the study, only one subject was positive for IgM antibodies by the gold labelling method. The positivity rate of IgM after recovery was very low, which was consistent with the relevant literature [15]. Therefore, we did not analyse the IgM data. (4) Data were not collected regarding basic disease information and laboratory parameters, such as leucocyte and lymphocyte counts, calcitonin sources, hypersensitive c-reactive protein or lymphocyte subsets (CD3, CD4, CD8, etc.), and an analysis of cellular immunity was not conducted.

## Conclusion

Based on this study of 87 patients with confirmed COVID-19 treated at our hospital and followed for 18 months, advanced age was associated with a higher risk of developing severe disease after infection. Specific IgG antibodies produced by natural infection were observed after 1 year. The change was less at 6 months, and the change at 1 year was 3.87 times than that at 6 months. The negative conversion rate of the severe and critical group was only 0.15 times than those of the mild and ordinary groups, and the duration of antibodies was longer. After stimulation by the new coronavirus vaccine, the IgG positivity rate was 100%, and the total antibody concentration was 2–4 times than that before the administration of the vaccine. The trend over time in total antibody levels was similar to that of IgG antibody levels. Neutralising antibodies following vaccination in patients who recovered from COVID-19 were two times more likely to inhibit the virus than those in vaccinated healthy controls. In conclusion, the risk of reinfection is lower in patients with previous COVID-19, and the vaccine is more protective in those individuals.

## Data Availability

The data used to support the findings of this study are available from the corresponding author upon request.

## Supplementary material

For supplementary material accompanying this paper visit https://doi.org/10.1017/S0950268822000528. click here to view supplementary material
