# Supplementary material for: Dynamics of specific antibodies in COVID-19 patients after recovery
Source: Epidemiol Infect. 2022 Mar 22;150:e70. doi: 10.1017/S0950268822000528 (PMC8987644; doi:10.1017/S0950268822000528)
Supplement: Supplementary file 1 [file S0950268822000528sup001.doc]

**Table S1.** Descriptive analysis of the total antibody concentration by disease group and time point.

**Table S2.** Effect analysis of time point trend.

**Table S3.** Comparison of groups defined by disease severity.

**Table S4.** Comparison of neutralizing antibody inhibition rates between the control group and experimental group (patient group).

**Table S1. Descriptive analysis of the total antibody concentration by disease group and time point.**

|  | **Disease group** | **Mean** | **Standard deviation** | Patients |
| --- | --- | --- | --- | --- |
| 1 month | Ordinary group | 242.253 | 275.1277 | 37 |
|  | Mild group | 285.2076 | 245.7159 | 40 |
|  | Severe (critical) group | 439.3989 | 229.845 | 10 |
|  | Total | 284.6627 | 261.0612 | 87 |
| 6 months | Ordinary group | 202.9598 | 231.3005 | 37 |
|  | Mild group | 249.1899 | 184.8811 | 40 |
|  | Severe (critical) group | 417.8618 | 232.3763 | 10 |
|  | Total | 248.9164 | 218.5741 | 87 |
| 12 months | Ordinary group | 115.3535 | 134.9303 | 37 |
|  | Mild group | 134.5069 | 124.0317 | 40 |
|  | Severe (critical) group | 237.8367 | 158.8357 | 10 |
|  | Total | 138.2382 | 136.4664 | 87 |
| After vaccination | Ordinary group | 427.0401 | 316.4476 | 37 |
|  | Mild group | 489.1061 | 266.8001 | 40 |
|  | Severe (critical) group | 518.0595 | 294.2382 | 10 |
|  | Total | 466.0382 | 290.6472 | 87 |

**Table S2. Effect analysis of time point trend.**

| **Disease severity group** | **(I) Point** | **(J) Point** | **Mean difference  (I-J)** | **Standard error** | **P** |
| --- | --- | --- | --- | --- | --- |
| Ordinary group | 1 month | 6 months | 38.968* | 12.465 | 0.0150 |
|  |  | 12 months | 126.147* | 25.904 | 0.0000 |
|  |  | After vaccination | -185.308* | 43.331 | 0.0000 |
|  | 6 months | 1 month | -38.968* | 12.465 | 0.0150 |
|  |  | 12 months | 87.179* | 18.587 | 0.0000 |
|  |  | After vaccination | -224.276* | 38.505 | 0.0000 |
|  | 12 months | 1 month | -126.147* | 25.904 | 0.0000 |
|  |  | 6 months | -87.179* | 18.587 | 0.0000 |
|  |  | After vaccination | -311.454* | 40.786 | 0.0000 |
|  | After vaccination | 1 month | 185.308* | 43.331 | 0.0000 |
|  |  | 6 months | 224.276* | 38.505 | 0.0000 |
|  |  | 12 months | 311.454* | 40.786 | 0.0000 |
| Mild group | 1 month | 6 months | 35.231* | 12.036 | 0.0260 |
|  |  | 12 months | 148.881* | 25.013 | 0.0000 |
|  |  | After vaccination | -205.157* | 41.841 | 0.0000 |
|  | 6 months | 1 month | -35.231* | 12.036 | 0.0260 |
|  |  | 12 months | 113.650* | 17.947 | 0.0000 |
|  |  | After vaccination | -240.388* | 37.181 | 0.0000 |
|  | 12 months | 1 month | -148.881* | 25.013 | 0.0000 |
|  |  | 6 months | -113.650* | 17.947 | 0.0000 |
|  |  | After vaccination | -354.038* | 39.383 | 0.0000 |
|  | After vaccination | 1 month | 205.157* | 41.841 | 0.0000 |
|  |  | 6 months | 240.388* | 37.181 | 0.0000 |
|  |  | 12 months | 354.038* | 39.383 | 0.0000 |
| Severe (critical) group | 1 month | 6 months | 25.886 | 24.814 | 0.8820* |
|  |  | 12 months | 211.627* | 51.567 | 0.0010 |
|  |  | After vaccination | -71.701 | 86.259 | 0.9570* |
|  | 6 months | 1 month | -25.886 | 24.814 | 0.8820* |
|  |  | 12 months | 185.741* | 37 | 0.0000 |
|  |  | After vaccination | -97.587 | 76.652 | 0.7500* |
|  | 12 months | 1 month | -211.627* | 51.567 | 0.0010 |
|  |  | 6 months | -185.741* | 37 | 0.0000 |
|  |  | After vaccination | -283.327* | 81.192 | 0.0050 |
|  | After vaccination | 1 month | 71.701 | 86.259 | 0.9570* |
|  |  | 6 months | 97.587 | 76.652 | 0.7500* |
|  |  | 12 months | 283.327* | 81.192 | 0.0050 |

**Table S3. Comparison of groups defined by disease severity.**

| **Point** | **Disease severity  group (I)** | **Disease severity group (J)** | **Mean difference  (I-J)** | **Standard error** | **P** |
| --- | --- | --- | --- | --- | --- |
| 1 month | Ordinary group | Mild group | -42.6580 | 59.0500 | 0.8530 |
|  |  | Severe/critical group | -200.1480 | 95.1940 | 0.1110 |
|  | Mild group | Ordinary group | 42.6580 | 59.0500 | 0.8530 |
|  |  | Severe/critical group | -157.4890 | 95.0920 | 0.2750 |
|  | Severe/critical group | Ordinary group | 200.1480 | 95.1940 | 0.1110 |
|  |  | Mild group | 157.4890 | 95.0920 | 0.2750 |
| 6 months | Ordinary group | Mild group | -46.3950 | 48.4870 | 0.7140 |
|  |  | Severe/critical group | -213.229* | 78.1650 | 0.0230* |
|  | Mild group | Ordinary group | 46.3950 | 48.4870 | 0.7140 |
|  |  | Severe/critical group | -166.8340 | 78.0810 | 0.1030 |
|  | Severe/critical group | Ordinary group | 213.229* | 78.1650 | 0.0230* |
|  |  | Mild group | 166.8340 | 78.0810 | 0.1030 |
| 12 months | Ordinary group | Mild group | -19.9240 | 30.4320 | 0.8860 |
|  |  | Severe/critical group | -114.6680 | 49.0590 | 0.0640 |
|  | Mild group | Ordinary group | 19.9240 | 30.4320 | 0.8860 |
|  |  | Severe/critical group | -94.7430 | 49.0070 | 0.1600 |
|  | Severe/critical group | Mild group | 114.6680 | 49.0590 | 0.0640 |
|  |  | Mild group | 94.7430 | 49.0070 | 0.1600 |
| After vaccination | Ordinary group | Mild group | -62.5080 | 67.0360 | 0.7300 |
|  |  | Severe/critical group | -86.5410 | 108.0670 | 0.8100 |
|  | Mild group | Ordinary group | 62.5080 | 67.0360 | 0.7300 |
|  |  | Severe/critical group | -24.0330 | 107.9520 | 0.9950 |
|  | Severe/critical group | Ordinary group | 86.5410 | 108.0670 | 0.8100 |
|  |  | Mild group | 24.0330 | 107.9520 | 0.9950 |

**Table S4: Comparison of neutralizing antibody inhibition rates between the control group and experimental group (patient group).**

|  |  | **Control group** | **Experimental group** | **χ2/t** | **P** |
| --- | --- | --- | --- | --- | --- |
| Sex | Male | 25 | 47 | 0.206 | 0.650 |
|  | Female | 25 | 40 |  |  |
| Age |  | 42.98±1.682 | 53.61±1.354 | 60.773 | 0.121 |
| Neutralizing antibody | Inhibition rate | 46.53±30.06 | 95.01±2.89 | -14.961 | 0.001 |
